# Tetrodotoxin poisoning with cardiorespiratory arrest and permanent neurological sequelae following ingestion of a checkered puffer *Sphoeroides testudineus* (Linnaeus, 1758) from the Brazilian Amazon coast

**DOI:** 10.1590/0037-8682-0544-2025

**Published:** 2026-06-15

**Authors:** Ingredy Eylanne Monroe Vidigal, Guilherme Vidigal Fernandes da Silva, Nívea Fernanda Maria Ferreira Costa, Marcillio Holanda Bezerra, Héllida Negrão Dias, Jamerson Aguiar Santos, Rachel Ann Hauser-Davis, Jorge Luiz Silva Nunes

**Affiliations:** 1 Universidade Federal do Maranhão, Programa de Pós Graduação em Biodiversidade e Biotecnologia da Amazônia Legal, São Luis, MA, Brasil.; 2 Universidade Federal do Maranhão, Laboratório de Organismos Aquáticos, Oceanografia e Limnologia, São Luís, MA, Brasil.; 3 Centro Universitário de Ensino Superior Dom Bosco, Departamento de Medicina, São Luís, MA, Brasil.; 4 Rede SARAH de Hospitais de Reabilitação, São Luís, MA, Brasil.; 5 Fundação Oswaldo Cruz, Laboratório de Avaliação e Promoção da Saúde Ambiental, Rio de Janeiro, RJ, Brasil.

Dear Editor:

Pufferfish, belonging to the Tetraodontidae family^1^, are considered a delicacy worldwide. Over 20 species of these fish store a potent neurotoxin, tetrodotoxin (TTX), in several tissues, including the epidermis, liver, spleen, and pancreas^2^ ^-^ ^4^.

In pufferfish, TTX is produced by endosymbiotic bacteria and can be toxic to humans. This neurotoxin is considered the most potent marine biotoxin associated with food poisoning cases^3^, with pufferfish ingestion comprising one of the most serious forms of aquatic animal poisoning, causing death within minutes if not treated^4^.

Patients preenting poisoning due to pufferfish ingestion have been routinely reported in Brazil, Singapore, Bangladesh, Japan, Australia, India, and the United States, among others, with cases in the latter being mainly due to pufferfish imported from Japan and Mexico and pufferfish mislabeled as other fishes^4^ ^,^ ^5^.

Pufferfish TTX is a non-protein, thermostable, water-soluble neurotoxin^4^ that causes muscle paralysis by blocking sodium channels^2^. The main symptoms of pufferfish poisoning are gastrointestinal, cardiovascular, and neurological in nature^4^. Neurological manifestations occur within a few hours after poisoning and include perioral and extremity paresthesia, muscle weakness, myalgia, vertigo, dysarthria, ataxia, difficulty walking, and visual disturbances. As neurological manifestations worsen, convulsions, dyspnea, and cardiorespiratory arrest may occur, usually within the first 24 h^4^.

To date, no specific treatment is available for pufferfish poisoning, and supportive treatment mainly comprises respiratory support. Immediate measures, such as gastric lavage and administration of emetics, may be indicated, although they are only effective in the first few hours after ingestion^4^. The prognosis of poisoning in humans is favorable if adequate supportive treatment is administered prior to the onset of cardiorespiratory arrest. However, this support is often delayed, leading to mortality or permanent morbidity. 

Although pufferfish are recognized as poisonous, only a few officially documented poisoning cases due to human consumption in Brazil have been reported^6^, most involving fishers and their families, who traditionally consume pufferfish meat, which contributes to the underreporting of these events^7^.

The scarcity of data on TTX poisoning makes it difficult to accurately assess the true extent of this disease in Brazil². In this context, this study describes a case of poisoning resulting from the ingestion of a pufferfish *Sphoeroides testudineus* (Linnaeus, 1758) (Figure 1) by an artisanal fisher on Maranhão Island, in the municipality of São Luís, Maranhão, Northern Brazil, and discusses other cases reported in the country.

**FIGURE 1:**
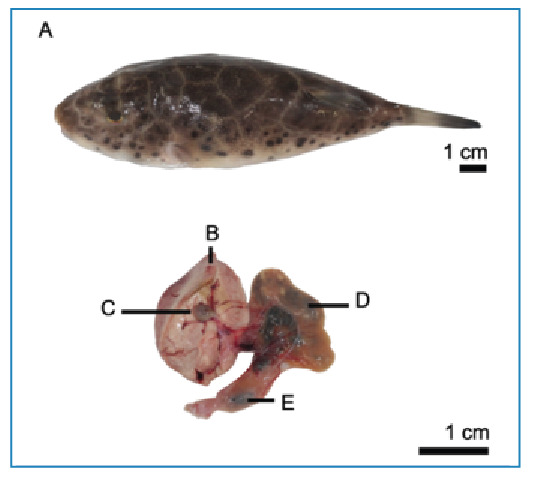
Top image: **(A)** Checkered pufferfish (Sphoeroides testudineus) used as a food source by the local population, similar to the fish consumed by artisanal fisher. Lower image: Viscera of Checkered pufferfish used as a food source by artisanal fishers. The highest concentration of tetrodotoxin is found in these organs. **(B)** Liver; **(C)** Gallbladder; **(D)** Stomach; **(E)** Intestine.

A 42-year-old artisanal fisher was poisoned after ingesting the liver of a checkered pufferh (*S. testudineus*). Clinical data were obtained through outpatient medical records and supplemented by an interview with the patient's legal representative, who provided complete clinical records from a multidisciplinary team covering the hospitalization period and specialized health care follow-up. Clinical, evolutionary, and therapeutic data are descriptively presented.

This study was approved by the Research Ethics Committee of the Federal University of Maranhão (no. 1.649.669, July 2016) and followed Brazil’s National Health Council guidelines (Resolution 466/12). A Free and Informed Consent Form was duly signed.

The poisoning took place in April 2017, when the artisanal fisher, after disembarking from a fishing trip, went to his residence and prepared the pufferfish liver for his own consumption (Figure 1), as on previous occasions. Based on the fish characteristics mentioned by the patient, we conclude that the consumed pufferfish species was *S. testudineus*, a common species in the region and routinely consumed by locals. Taxonomic confirmation was performed through specialized literature.

About 10 min after ingesting the pufferfish, the patient presented hyperemia and limb paresis, accompanied by convulsions. The patient’s wife rescued him, and he was taken to a local emergency hospital. At the hospital, he showed signs of improvement, with cardiorespiratory arrest reversed after 7 min. The patient was transferred to the intensive care unit, where he remained for 12 days. 

A brain magnetic resonance imaging (MRI) evaluation revealed a hypersignal on T2/FLAIR in the diagnostic impression, affecting both the globus pallidus with marked low-signal *foci* and a low SWI sequence signal, probably associated with microhemorrhage areas. In addition, a sign of laminar cortical necrosis was noted in the cortex of the mesial face of the right occipital lobe, potentially related to severe hypoxic-ischemic damage. Moderate ectasia of the suprasensory system was identified as a compensatory mechanism. A cranial tomography and MRI demonstrated an image of post-arrest infarction with involvement of the right occipital region and the globus pallidus bilaterally following arrest.

During hospitalization, the patient presented infectious complications that required antibiotic administration, including ceftriaxone, clindamycin 600 mg, and oxacillin 50 mg.

The patient was discharged with cognitive sequelae, partial blindness, limb paresis, difficulty in locomotion, retrograde amnesia, and total dependence concerning basic daily life habits. The artisanal fisher was referred for follow-up with motor physiotherapy, speech therapy, occupational therapy, consultations with an ophthalmologist and nutritionist specialist, and prescribed the following medications: ranitidine 150 mg (one tablet PO every 8 h), diazepam 10 mg (v tablet PO every 12 h), risperidone 2 mg (one tablet PO every 12 h), haloperidol 5 mg (one tablet PO every 12 h), and melatonin 5 mg (one tablet PO 1 h before bedtime).

To aid in poisoning identification and healthcare management, Fukuda and Tani^8^ developed a symptom grading scale for clinical TTX poisoning in 1941^8^, highlighting the degree of involvement according to symptom onset^5^ (Table 1).

**TABLE 1:** Tetrodotoxin poisoning symptom, based on a scale created by Fukuda and Tani in 1941.

Degree	Symptoms	Time to symptom
I	Oral paresthesia/numbness with or without nausea	5-45 min
II	Diffuse paresthesia/numbness, motor paralysis of extremities, mild ataxia, slowed speech and normal tendon reflexes.	10-60 min
III	Severe ataxia, aphonia, dysphagia, dyspnea, cyanosis, hypotension, fixed or dilated pupils, chest pain, but preserved consciousness	15 min to several hours
IV	Coma, severe respiratory failure, hypoxia, hypotension, bradycardia, and arrhythmia.	15 min to several hours

The symptoms triggered by poisoning in this report were classified as grade IV, with tetraparesis, respiratory failure, and cardiorespiratory arrest, consequently causing hypoxic-ischemic lesions in the brain and permanent patient morbidity.

Studies have highlighted poisonings caused by the ingestion of the viscera and liver of freshwater pufferfish in Brazil^4^ ^,^ ^7^, including one case in which a 2-year-old child died following the ingestion of pufferfish viscera, presenting cold sweats, progressive muscle weakness, and cardiorespiratory arrest, culminating in death. In another case, the patient was discharged from the hospital without sequelae due to immediate medical support. In both cases, no neurological sequelae were noted, in contrast to the present study.

Patients presenting poisoning by spotted pufferfish have been recorded in 11 members of the same family, with initial paresthesia symptoms in the perioral region and nausea/vomiting^7^. Of these, three patients were severely affected (one adult and two children) and were treated in intensive care units. The adult patient suffered a cardiac arrest, and one child presented mydriasis, albeit with response to light stimulation. Both were diagnosed with respiratory failure and received mechanical ventilatory and therapeutic clinical support, which constitutes a critical situation in this type of accident. All patients showed improvement in their clinical condition and were discharged^7^.

Thus, the implementation of appropriate and timely healthcare procedures is crucial to prevent patient complications and death. Early symptom recognition coupled with rapid medical intervention, such as supportive care, respiratory assistance, and toxin management, can significantly reduce the risk of severe outcomes. Given the neurotoxic nature of pufferfish poisoning, delaying treatment can lead to irreversible damage, including paralysis and respiratory failure, which may be fatal. Adequate healthcare provider training, especially in coastal regions where pufferfish consumption is more common, is essential to ensure swift diagnosis and treatment, minimize the impact of pufferfish poisoning, and improve patient survival rates^4^. Educational campaigns to reduce consumption and recommendations for seeking health services should also be implemented.

## Data Availability

Research data are available upon reasonable request.
